# Evaluation of Federated Learning in Phishing Email Detection

**DOI:** 10.3390/s23094346

**Published:** 2023-04-27

**Authors:** Chandra Thapa, Jun Wen Tang, Alsharif Abuadbba, Yansong Gao, Seyit Camtepe, Surya Nepal, Mahathir Almashor, Yifeng Zheng

**Affiliations:** 1 Commonwealth Scientific and Industrial Research Organisation, Data61, Sydney 2122, Australia; 2 School of Chemical Engineering, The University of New South Wales, Sydney 2052, Australia; 3 Cyber Security Cooperative Research Centre, Australian Capital Territory 2604, Australia; 4 Harbin Institute of Technology, Harbin 150001, China

**Keywords:** federated learning, phishing email detection, recurrent neural network, bidirectional encoder representations from transformers (BERT)

## Abstract

The use of artificial intelligence (AI) to detect phishing emails is primarily dependent on large-scale centralized datasets, which has opened it up to a myriad of privacy, trust, and legal issues. Moreover, organizations have been loath to share emails, given the risk of leaking commercially sensitive information. Consequently, it has been difficult to obtain sufficient emails to train a global AI model efficiently. Accordingly, privacy-preserving distributed and collaborative machine learning, particularly federated learning (FL), is a desideratum. As it is already prevalent in the healthcare sector, questions remain regarding the effectiveness and efficacy of FL-based phishing detection within the context of multi-organization collaborations. To the best of our knowledge, the work herein was the first to investigate the use of FL in phishing email detection. This study focused on building upon a deep neural network model, particularly recurrent convolutional neural network (RNN) and bidirectional encoder representations from transformers (BERT), for phishing email detection. We analyzed the FL-entangled learning performance in various settings, including (i) a balanced and asymmetrical data distribution among organizations and (ii) scalability. Our results corroborated the comparable performance statistics of FL in phishing email detection to centralized learning for balanced datasets and low organizational counts. Moreover, we observed a variation in performance when increasing the organizational counts. For a fixed total email dataset, the global RNN-based model had a 1.8% accuracy decrease when the organizational counts were increased from 2 to 10. In contrast, BERT accuracy increased by 0.6% when increasing organizational counts from 2 to 5. However, if we increased the overall email dataset by introducing new organizations in the FL framework, the organizational level performance improved by achieving a faster convergence speed. In addition, FL suffered in its overall global model performance due to highly unstable outputs if the email dataset distribution was highly asymmetric.

## 1. Introduction

Email is the most common means of formal communication. At the same time, it is also exploited as a common attack vector for phishing attacks, where attackers disguise themselves as trustworthy entities and attempt to install malware or obtain sensitive information, such as login credentials and bank details of an email recipient. Based on the 2019 phishing and email fraud statistics [1], phishing accounted for 90% of data breaches, which led to an average financial loss of USD 3.86M. Moreover, phishing attacks cost American businesses half a billion dollars per year [2], and this activity has increased. Recently, COVID-19 drove phishing emails to an unprecedented level of over 600% increase [3].

To protect users from phishing attacks, various techniques have been devised. These techniques can generally be divided into two categories, traditional methods and artificial intelligence (AI)-based methods. Traditional methods have relied on known email formats, which are inefficient because attackers can easily manipulate email formats over time. In comparison, the AI-based methods are context-aware. These methods can continuously learn from newly available emails and adapt to handle the new attack formats/cases efficiently.

Among AI-based methods, deep learning (DL) feeds the email data directly into the system without requiring delicate feature engineering. Moreover, feature engineering is usually a time-consuming and laborious domain-specific task necessary for conventional ML-based methods, such as decision trees [4]. This makes DL a suitable method for learning against evolving threats over time. Convolutional neural network [5], recurrent convolutional neural network (RCNN) [6], and transformers [7] are typical examples of DL-based methods. Although DL-based methods are preferable to other methods considering their performance and automated feature engineering, they require a considerable amount of email data to be effective.

Unfortunately, organizations (referred to as clients in the remainder of this paper) often consider emails private, and disclosure to third parties has often been avoided [8]. Even anonymization of emails has been problematic because it can be easily circumvented, as attackers can exploit various characteristics, e.g., social graphs, to re-identify the victim’s entity [9]. As a result, it has been difficult to aggregate emails for centralized analysis. In addition, a recent work [10] emphasized the significant ethical concerns when accessing and analyzing the emails of 92 organizations, even with access permission. For any purpose, improperly centralized data management could violate specific rules, such as reusing the data indiscriminately and risk-agnostic data processing [11], which is required by the general data protection regulation (GDPR) [12] and HIPAA [13]. Therefore, even with user permission to use their data for agreed-upon tasks (e.g., DL), handling the email data in a centralized cloud is still challenging under the set of privacy regulations. Therefore, there is an urgent need for methods that preserve data privacy in DL and break the email data silos. As such, DL can access abundant email datasets and improve its performance (e.g., detection accuracy).

In this regard, federated learning (FL) [14,15,16], the most popular collaborative learning, is a suitable candidate method. It trains a joint DL model by harvesting the rich distributed data held by each client in a default privacy mode. The privacy of the raw data is enabled by two means; firstly, the data are never shared with other clients/participants, and, secondly, the data are always within the control of the data custodians (i.e., clients). Consequently, the data custodians have an assurance of some level of privacy and control over their data, motivating them to participate in distributed machine learning for the overall social good (e.g., an anti-phishing DL model with high detection accuracy).

FL has been explored in various applications, such as finance [17], health [16,18], and natural language processing (NLP) [19]. However, it is still unclear how efficient and effective it would be in phishing email detection with regard to the relevant deep models, such as (i) THEMIS [6], which has been the best-performing RCNN-based centralized model on phishing emails, and (ii) bidirectional encoder representations from transformers (BERT) [7], which has been a popular transformer-based centralized model on text data. Leveraging FL on email data is a similar concept to using FL in NLP, but the challenge here is that phishing emails are highly subjective, e.g., spear phishing, and email datasets are typically smaller than an NLP corpus.

To the best of our knowledge, the applicability of FL for detecting phishing emails had not been explicitly investigated. Therefore, this work took the first studies of FL-based phishing email detection and investigated its performance using DL models. Furthermore, we performed detailed studies of FL-based phishing detection by asking six research questions, including its performance and other aspects, such as (1) the number of distributed clients (also the data sources) and its effects on FL performance, (2) communication overhead, (3) increasing the client-level local model performance, and (4) data distribution among the clients and its effects on overall learning. These studies provides insights into the practical aspects of FL-based phishing detection and its development.

### Our Contributions

This work considered both the balanced and asymmetrical data distribution among clients. The evaluations were carried out with the following six research questions in mind.

RQ1 (Balanced data distribution) Could FL be applied to learn from distributed email repositories to achieve comparable performance to centralized learning (CL)?
  We developed deep-learning phishing email detection models based on FL and CL considering a balanced data distribution. Their performances demonstrated that FL achieved a comparable performance to CL. For example, (i) at epoch 45, THEMIS had 99.3% test accuracy in CL and 97.9% global test accuracy in FL with 5 clients; and (ii) at epoch 15, BERT had 96.2% test accuracy in CL and 96.1% global test accuracy in FL with 5 clients. The details are provided in Section 4.1.
RQ2 (Scalability) How would the number of clients affect FL performance and convergence?
  Our experiments considering a balanced data distribution suggested that, while keeping the same total email dataset, the convergence of the accuracy curve, and its maximum value was model dependent. We observed that THEMIS decreased by around 0.5% on global test accuracy at epoch 45 when increasing the number of clients from 2 to 5 in FL; however, BERT improved by around 0.6% on global test accuracy at epoch 15 when increasing the number of clients from 2 to 5. The details are provided in Section 4.1.
RQ3 (Communication overhead) What would the communication overhead be resulting from FL?
  FL had a communication overhead as a trade-off to privacy, and it was only dependent on the model size. For example, we quantified the overhead per global epoch per client for THEMIS at around 0.192 GB, and for BERT, at around 0.438 GB, for all cases in our settings. We regard such overheads as not of particular concern for organizational-level participants. The details are provided in Section 4.1.
RQ4 (Client-level perspectives in FL) Could a client leverage FL to improve its performance?
  We investigated client-level performances considering both balanced and asymmetrical data distributions in FL, including the cases where clients were available over time in the training process, and the total email dataset increased with the addition of new clients. A fast convergence in the accuracy curve was observed with THEMIS. The details are provided in Section 4.2.
RQ5 (Asymmetric data distribution) How would FL perform considering asymmetric data distributions among clients due to the variations in local dataset sizes and local phishing to legitimate sample ratios?
  Our studies of THEMIS with 2, 5, and 10 clients demonstrated that FL performed well and similarly in asymmetric data distributions due to the differences in local dataset sizes and the local phishing to legitimate sample ratios. Therefore, FL was resilient in these scenarios. The details are provided in Section 4.3.
RQ6 (Asymmetric data distribution) How would FL perform under extreme dataset diversity among clients?
  Data asymmetry, in this case, was due to the class skewness and different dataset sizes (small to large) across the clients. Our studies illustrated that forming a best-performing global model for all clients under FL was not straightforward. In addition, the local and global performances were model dependent. The details are provided in Section 4.4.

## 2. Background

### 2.1. Centralized Learning

Centralized learning (CL) is typically performed by aggregating all available datasets (e.g., phishing and legitimate emails) at one central repository. Then it performs centralized machine learning on the aggregated dataset. During the learning process, a modeler can access the raw data shared by one or more clients, thus making it unsuitable if the data are private, such as emails. In addition, in the era of big data and deep learning, it is non-trivial to maintain the required resources, including storage and computation, in CL. Therefore, there is a rise in distributed learning, particularly federated learning.

### 2.2. Federated Learning

**Figure 1:**
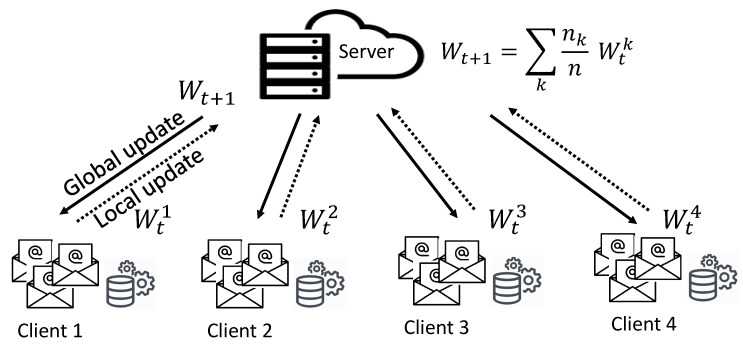
An overview of federated learning.

Federated learning (FL) [14] allows parallel deep learning training across distributed clients and pushes the computation to the edge devices (i.e., clients). Figure 1 illustrates an overview of FL with one coordinating server and four exemplified clients having their local email datasets. Firstly, each client *k*, k∈{1,2,3,4}, trains the model on their local email dataset $D_{k}$ and produces the local model $W_{t}^{k}$ at time instance *t*. Secondly, all clients upload their local models to the server. Then the server performs the weighted averaging (i.e., aggregation) of the local models and updates the global model $W_{t+1}$. Finally, the global model is broadcast to all clients (model synchronization), and this completes the one round, known as one global epoch, of the FL process. This process continues until the model converges (see Algorithm 1). In FL, the server synchronizes the training process across the clients. Over the entire training process, only the models (i.e., model parameters) are transmitted between the clients and the server. Therefore, a client (e.g., financial institution) does not require sharing their raw email data with the server (e.g., coordinated by an email analyzer) or any other clients during the training process. Therefore, the data are always local and kept confidential in FL.
**Algorithm 1:** Federated learning 
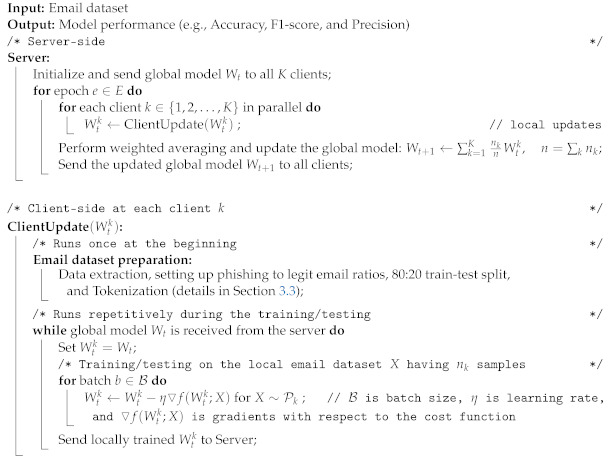


## 3. Experimental Setup

### 3.1. Datasets

In this study, primarily, phishing and legitimate email samples were collected from three popular sources, namely first security and privacy analytics anti-phishing shared task (IWSPA-AP) [20], Nazario’s phishing corpora (Nazario) [21], and Enron email dataset (Enron) [22]. In addition, we considered phishing emails from CSIRO (private emails, https://www.csiro.au/ accessed on 3 January 2021) and Phishbowl [23]. The dataset contains email samples with (i) header (email header precedes the email body and contains information of the header fields, including To, Subject, Received, Content-Type, Return-Path, and Authentication-Results), and (ii) without header; IWSPA-AP has both types, whereas all email samples in Nazario and Enron have the header accompanied by the body, while there is no header for CSIRO and Phishbowl emails. Overall, the data sources include Wikileaks archives, SpamAssassin, IT departments of different universities, synthetic emails created by Data engine [24], Enron (emails generated by employees of Enron corporation), Nazario (personal collection), and private emails (CSIRO). CSIRO emails are phishing emails reported by CSIRO staff between 2017 and 2020, and we manually labeled them to remove the spam. Phishbowl emails are published by Cornell University, and we collected emails reported from April 2019 to January 2021. The emails on the website have a header but with partial fields or body only, so we considered the body only of these emails for our dataset. To provide more insight into the email samples, we present some frequently appearing words in them as follows:

- IWSPA-AP phishing emails (a) with header include “account”, “PayPal”, “please”, “eBay”, “link”, “security”, “update”, “bank”, “online”, and “information”; and (b) without header include “text”, “account”, “email”, “please”, “information”, “click”, “team”, “online”, and “security”. IWSPA-AP legitimate emails (a) with header include “email”, “please”, “new”, “sent”, “party”, “people”, “Donald”, “state”, and “president”; and (b) without header include “text”, “link”, “national”, “US”, “Trump”, and “democratic”.
- Nazario includes “important”, “account”, “update”, “please”, “email”, “security”, “PayPal”, “eBay”, “bank”, “access”, “information”, “item”, “click”, “confirm”, and “service”.
- Enron includes “text”, “plain”, “subject”, “please”, “email”, “power”, “image”, “time”, “know”, “this”, “message”, “information”, and “energy”.
- CSIRO includes “shopping”, “parcel”, “invitation”, “payment”, “employee”, “webinar”, “survey”, “newsletter”, “program”, and “workshop”.
- Phishbowl includes “account”, “id”, “password”, “Cornell”, “upgrade”, “notice”, “administrator”, “message”, “job”, “server”, and “verify”.

We considered the updated email dataset from the sources, e.g., Nazario’s phishing corpus 2019. In total, the experimental dataset had 23,916 email samples, and Table 1 presents the number of emails extracted from each source.

### 3.2. Deep Learning Model Selection

We selected two models that are described in the following.

#### 3.2.1. THEMIS

THEMIS is one of the recent models which has been demonstrated to be highly effective for phishing email detection. It employs recurrent convolutional neural networks (RCNNs) and models emails at multiple levels, including char-level email header, word-level email header, char-level email body, and word-level email body [6]. This way, it captures the deep underlying semantics of the phishing emails efficiently. Consequently, it makes THEMIS better than existing DL-based methods that are limited to natural language processing and deep learning [5].

##### Overview of THEMIS

**Figure 2:**
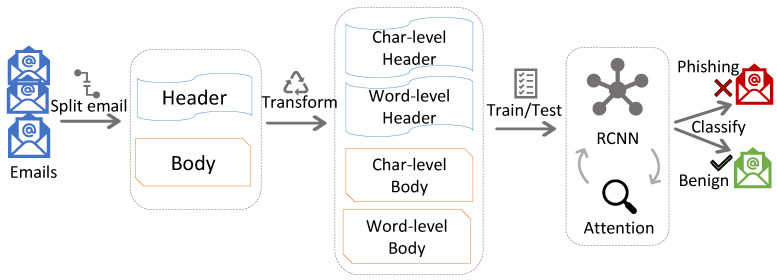
An overview of THEMIS for phishing detection.

Figure 2 illustrates a system overview of THEMIS for phishing detection. Firstly, THEMIS extracts the char level and word level of the email header and body, and then an embedding layer converts all these levels to the respective vector representation. Afterward, it feeds each vector representation into the RCNN [25] and learns a representation for the email header and email body, respectively. THEMIS RCNN consists of four bidirectional-long short-term memory (Bi-LSTM) that obtain the left and right semantic information of a specific location with its embedding information from the above four vectors, thus forming something called a triple. Next, these triples are mapped into specified dimensions using a tanh activation function. The longitudinal max polling is then applied to obtain four different representations, which will be paired to form only two representations for the header and the body. As the email header representation and body representation have varying degrees of impact on phishing detection, an attention mechanism is applied to compute a weighted sum of the two representations. This produces an ultimate representation of the whole email, which is further processed to produce the classification result. For more details on THEMIS, we refer readers to [6].

The THEMIS original paper considered only emails with headers (8780 samples), and THEMIS was trained on that data [6]. In contrast, our study considered (i) around 2.7× more email samples, (ii) emails with header and without header information, (iii) THEMIS under emails with both headers and without headers (body only), and we analyzed its performances for CL and FL.

#### 3.2.2. Bidirectional Encoder Representations from Transformers

Bidirectional encoder representations from transformers (BERT) [7] is a language model initially developed by Google. Transformer encoders are basic blocks of BERT. Transformers learn the contextual information in the input sequence by an attention mechanism that enables them to relate different parts of the input sequence to find their relationship; for example, the contextual information of the words/sub-words in a sentence. BERT reads the entire input sequence to become bidirectionally trained. This enables BERT to learn the contextual information better than the techniques looking at the sequence from one direction (e.g., each word conditioned on its previous or next words).

##### Overview of BERT

**Figure 3:**
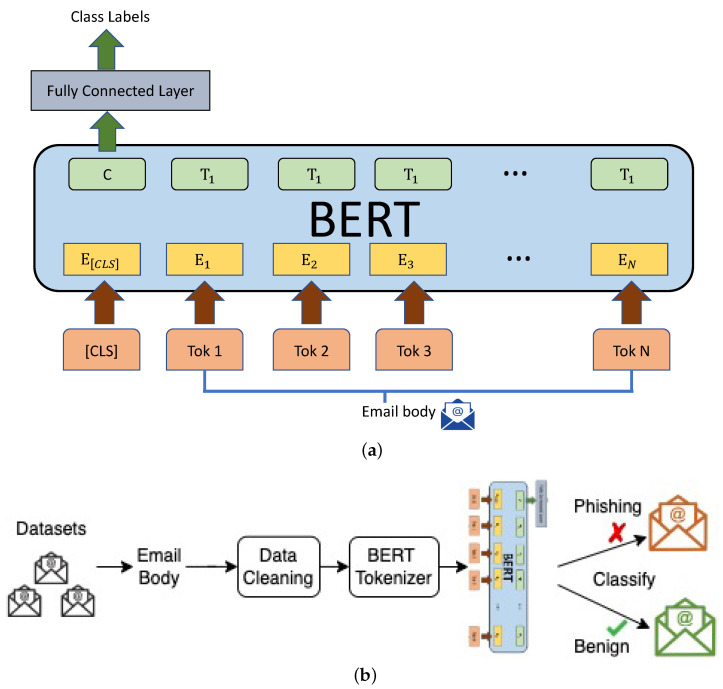
An overview of BERT for phishing detection, (**a**) BERT model, where E is input embedding, C and $T_{i}$ are the final hidden vectors of token [CLS] and *i*th token, respectively, and (**b**) overall process.

In this study, we used Huggingface’s library called transformers to use the bert-base-uncased model pre-trained on a large corpus of English texts [26], such as English Wikipedia and BookCorpus. The model has 12 layers of transformers, 768 hidden sizes, 12 self-attention heads, and altogether 110 million parameters. BERT has been used for various tasks other than natural language processing by performing its fine-tuning. In this process, a few layers are added to the end of the model (e.g., classification layer) and train/test the whole model with a small learning rate on the available dataset. The model was pre-trained for two tasks, masked language modeling (MLM) and next sentence prediction (NSP). MLM predicted the masked words in a sentence whose 15% of words were randomly masked by the model at the beginning. NSP predicted whether or not two sentences, which had their words masked to some percentage, followed each other. Therefore, the embedding had special tokens (i) [CLS] at the beginning of each sentence, (ii) [SEP] to separate two sentences in a sequence and at the end of the sentence, and (iii) [MASK] to mask any word in the sentence. An overview of BERT for a classification task is depicted in Figure 3.

BERT was used in phishing email detection [27]. The authors designed a smaller BERT, called CatBERT, by pruning odd-numbered transformers from it and replacing those with adapters. CatBERT achieved an 87% detection rate on their own data collected at Sophos, and it had the best performance compared to the DistilBERT (compressed BERT) [28] and LSTM-based models on their dataset. Our focus in this study was to demonstrate the feasibility of FL on the detection side. We explored the standard BERT rather than distilled BERT (which only benefits computation). To our best knowledge, only CL has been performed and analyzed for BERT in phishing email detection. Moreover, in CL, the performance of the standard BERT in phishing email detection is still unclear. In our experiments, BERT considered only the body of email samples as input because, unlike THEMIS, it has no dedicated architectural part to obtain all header fields separately; however, it is possible to concatenate the header information to the body and feed it to BERT.

### 3.3. Data Preparation

Our FL setups had different data sources and data distribution among the clients. For RQ1 to RQ5, we considered three email sources, namely IWSPA-AP, Nazario, and Enron, with a total of 23,475 email samples. We considered the other two sources, CSIRO and Phishbowl emails, for RQ6 (refer to Table 2). We did this division as there were only 441 phishing email samples from CSIRO and Phishbowl, and they were analyzed only in the extreme dataset diversity.

In our balanced dataset setup for RQ1 to RQ4, including the asymmetric dataset for RQ4, we considered equal phishing and legitimate email samples out of 23,475 data samples. So, we prepared the experimental dataset of size 20,044 (i.e., 2 × 10,022); to be precise, 10,022 was aligned with the number of phishing emails while the number of legitimate emails was 13,453. Moreover, the new dataset had four parts—phishing header, phishing body, legitimate header, and legitimate body—each with 10,022 samples. The experimental dataset was equally and uniformly distributed in all our distributed setups with multiple clients except for the cases with the asymmetric dataset (RQ4, RQ5 and RQ6). For example, cases with five clients had a dataset of size 4008 (i.e., around 20,044 divided by 5) in each client. In the distributed setup for RQ5, we performed two experiments (i) each client had different sizes of email samples with a 50:50 phishing to legitimate email ratio, and (ii) each client had the same sizes of email samples but with different phishing to legitimate email ratio. In RQ6, we considered five clients, each uniquely corresponding to one of our five data sources.

We performed all the above data distributions for experimental setups, which simulated cases with multiple organizations having their own local data (distributed data) in different geo-locations and remaining in silos. For all experiments, the training-to-testing data split ratio was 80:20.

Our email data sources had two file formats, text file (.txt) and mbox file (.mbox). Each email was a single text file if the email sample was in text format. In the mbox format, all messages were concatenated and stored as plain text in a single file. Moreover, each message started with the four characters "From" followed by a space. Both types of email files were parsed into two parts, namely email header and email body, and then subjected to further processing, including cleaning and tokenization.

#### 3.3.1. Extraction of Header and Body

The class Header of the Python module, called email.header [29], was used to extract the email header, and this separated the header and body part of the email samples. In the header section, we considered only the “Subject” and the “Content-Type” fields, deemed essential for phishing detection. This separation was performed using the Python library called the regular expression (RE) module [30].

#### 3.3.2. Cleaning of the Extracted Header and Body

The Python library Beautiful Soup 4 [31] and HTML parser [32] were used to clean the text information in HTML format. In addition, we used RE for cleaning the plain text (both in the header and body) by removing punctuation and non-alphabetic characters. To filter out the stop words from the header and body, we used stopwords of the nltk packages (nltk.corpus) [33] of Python.

#### 3.3.3. Tokenization

Our two models under investigation required different tokenization methods. For THEMIS, we performed tokenization in the following way. To obtain the char-level and word-level sequences of the tokens for both header and body parts, the tokenizer class provided by Keras library [34] was used. This was to encode each character/word as a unique integer as required by the input format of the embedding layer. In addition, two main functions were used for tokenization, ‘fit_on_texts,’ which updated internal vocabulary based on a list of texts, and ‘texts_to_sequences,’ which transformed each text in texts to a sequence of integers by considering only words known by the tokenizer. In all our measurements, we kept 50, 100, 150, and 300 as the length of the four sequences of tokens, word-level header, char-level header, word-level body, and char-level body, respectively.

For BERT, we considered only the email body and performed its tokenization in the following way. We used BertTokenizer [35], provided by the Huggingface library, to encode the email’s body to tokens. In addition, the tokenizer inserted additional special tokens, such as [CLS] and [SEP]. BERT allowed only 512 tokens to be inputted at a time, and it was considered during tokenization. Furthermore, the tokenizer returned original input ids, attention masks, and token type ids required during learning.

### 3.4. Experimental Steps

For performance analysis, we used a high-performance computing (HPC) platform built on Dell EMC’s PowerEdge platform. It had the Tesla P100-SXM2-16GB GPU model. All code was written in Python 3.6.1. THEMIS, which has an RCNN, was implemented using TensorFlow 2.2.5 [36] and Keras 2.2.5 [37] framework, and BERT, a pre-trained transformer model, was downloaded from the Huggingface library. In all measurements, we kept the same random seed, i.e., random.seed(123). We ran centralized and federated model training in various settings but with the same (i) learning rate of 0.0001 and batch size of 256 for THEMIS and (ii) learning rate of 0.00001 and batch size of 4 for BERT. The specific batch size was chosen based on the available resources (e.g., GPU had 16 GB internal memory). The overall processes for THEMIS and BERT for phishing email detection are depicted in Figure 2 and Figure 3, respectively. The email dataset setup based on research questions (RQs) is summarized in Table 2.

## 4. Results

To ease the presentation, we divide this section into four parts, where the empirical results for six research questions are presented. As we performed experiments with THEMIS, considering both with and without email header information, for convenience, we refer to (i) THEMIS if we consider both email’s header and body information and (ii) THEMISb if we consider only the email’s body information in the remainder of this paper.

### 4.1. Distributed Email Learning with Balanced Data Distribution

Considering the CL’s accuracy as the baseline for RQ1, RQ2, and RQ3, we performed experiments in a balanced data distribution among the clients where the total dataset remained the same. In other words, for the total dataset *D*, and for any number of clients K≥1, ${⋃}_{k}D_{k}=D$, where $D_{k}$ is the dataset of the client *k*, k∈{1,2,⋯,K}. We kept the same size of the total dataset despite the change in the number of clients. This was performed to see the effect of the change in the number of clients (datasets distribution) within the same total dataset. In our setups, we reasonably assumed that the clients were with resourceful computation to jointly train the FL model to preserve the privacy of emails.

**Figure 4:**
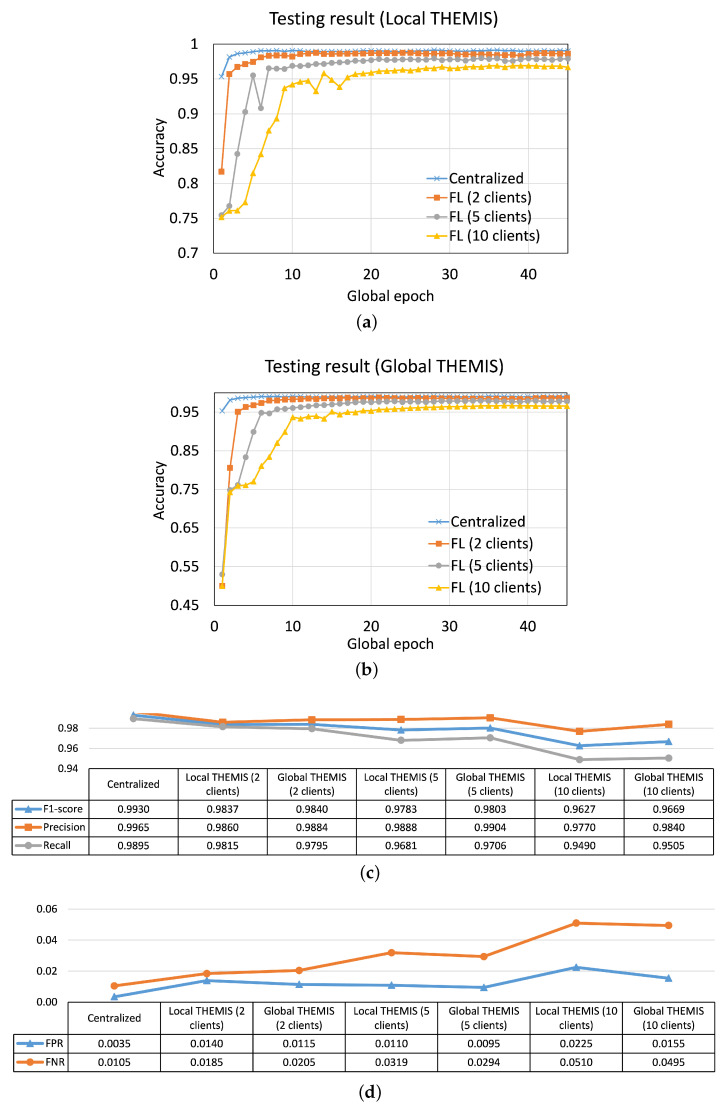
Results for THEMIS: Convergence curves of average testing accuracy for (**a**) local models and (**b**) global model. Performance metrics of testing results at global epoch 45 in (**c**) centralized learning and (**d**) federated learning (FL) with 2, 5, and 10 clients.

**Figure A1:**
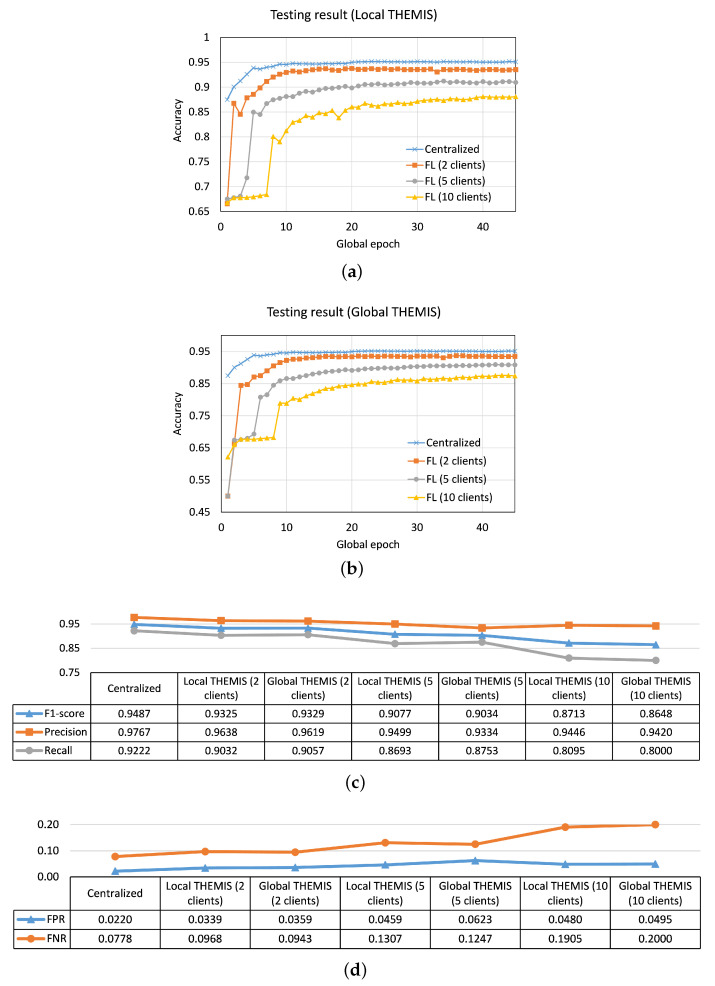
THEMISb and federated learning (FL): Convergence curves of average testing accuracy for (**a**) local models and (**b**) global model considering body only of email samples. Corresponding performance metrics of the testing results at global epoch 45 in (**c**) centralized learning and (**d**) FL with 2, 5, and 10 clients.

How Did THEMIS and THEMISb Perform?THEMIS outperformed THEMISb in our empirical results. In CL, at global epoch 45, for THEMIS, we observed accuracy of 99.301%, FPR of 0.0035, and FNR of 0.0105 (see Figure 4), whereas THEMISb only provided an accuracy of 95.085% (dropped by around 4%), FPR of 0.022 and FNR of 0.0778 (see Figure A1). This indicates that header information was critical for THEMIS, and it was leveraging them well for phishing detection. The accuracy and FPR stated in the THEMIS paper [6] were 99.848% and 0.043%, respectively. These values were nominally different than our case. This can be due to various reasons, including email data samples, sample size (see Section 3.2.1), and model hyper-parameters.

RQ** 1.** *Could FL be applied to learn from distributed email repositories to achieve comparable performance to centralized learning (CL)?*

**Figure 5:**
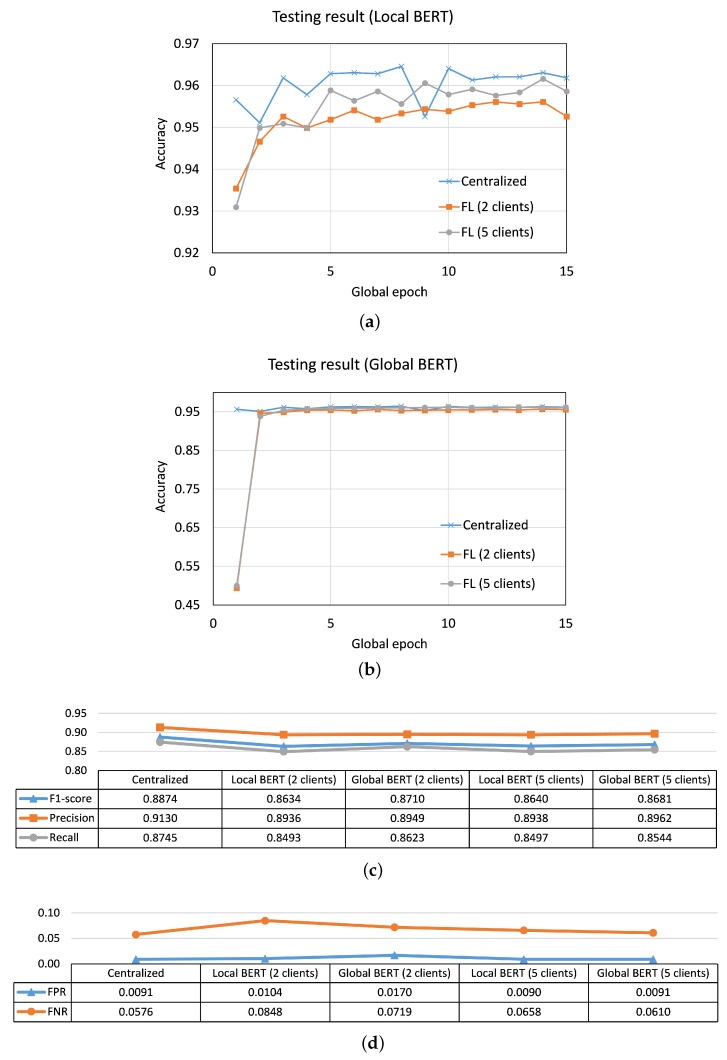
Results for BERT: Convergence curves of average testing accuracy for (**a**) local models and (**b**) global model. Corresponding performance metrics of the testing results at global epoch 15 in (**c**) centralized learning and (**d**) federated learning (FL) with two and five clients.

In FL with 2, 5, and 10 clients, THEMIS converged to obtain local and global model test accuracy greater than 96% in the observation window of 45 global epochs. However, none achieved the CL performance of 99.3% accuracy within the observation window. A similar performance was observed for THEMISb. For BERT (which only considered emails without header information while training/testing), at global epoch 15, we had (i) in CL, testing accuracy of 96.183%, FPR of 0.0091, and FNR of 0.0576, (ii) in FL with two clients, global testing accuracy of 95.559%, FPR 0.017 of and FNR of 0.0719, and (iii) in FL with five clients, global testing accuracy of 96.11%, FPR 0.0091 of and FNR of 0.0610 (see Figure 5). We found that the performance of BERT was not good as THEMIS, but it was better than THEMISb in our results.


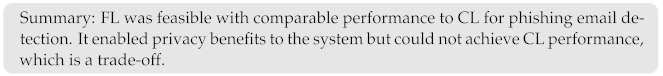

RQ** 2.** *How would the number of clients affect FL performance and convergence?*

The convergence patterns for both the local and global models were similar for THEMIS in FL. However, in both cases, the average performances degraded with an increase in the number of clients. For example, the global testing accuracy had dropped by 1.8% at global epoch 45 when increasing the number of clients from 2 to 10 (see Figure 4). The potential reason for this drop might be the effect on the convergence rate due to local shuffling in distributed setup; the convergence rate was dominated by local training dataset size, and more data is better for an overall performance [38]. For THEMISb, the drop was about 6% when increasing the number of clients from 2 to 10. This drop in THEMISb was significant in comparison to THEMIS.

For BERT in FL, the local and global performance drop was negligible compared with the BERT’s performance in CL, e.g., only 0.6% for 2 clients (see Figure 5). In contrast to THEMIS/THEMISb performance, the BERT performance did not degrade with the increase in the number of clients. BERT in FL with 5 clients showed better performance (0.6% increase in accuracy at global epoch 15) than BERT in FL with 2 clients.


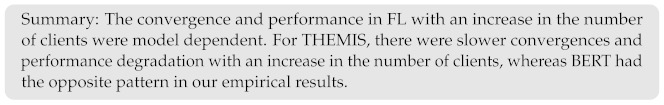

RQ** 3.** *What would the communication overhead be resulting from FL?*

As the main server was assumed to have sufficient resources to handle any communication overhead, our concern was focused on clients with relatively low resources than the server. Therefore, the quantification of communication overhead in FL was limited to the client side. We measured the data uploaded (i.e., a sum of the data packet size of $W_{t}^{k}$ and $n_{k}$) and downloaded (i.e., the data packet size of $W_{t+1}$) to and from the server, respectively, and the results were averaged by the total number of the clients. In CL, we did not consider a client-server setup; therefore, the communication overhead was zero.

As the sample size information was negligible compared to the model size, the size of data downloaded and uploaded was almost the size of the global and local models, respectively, while training at each client in FL. Therefore, the communication overhead depended solely on the model size, not the number of clients or epochs. Our results with the various number of clients verified this. For THEMIS and BERT, we observed a consistent average communication overhead of around 0.192 GB and 0.438 GB per global epoch per client, respectively, for all cases. A well-connected setup with wired or wireless connections between the server and clients can easily address the overhead. Therefore, this is not a concern for organizational-level participation (assumed to have sufficient computing resources) in distributed phishing email learning.


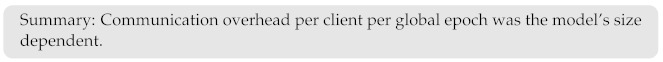


### 4.2. Client-Level Perspectives in Federated Learning

To demonstrate the client-level performance in phishing email detection in distributed setups, we performed three studies in a balanced and asymmetric data distribution among the clients, where the total dataset changed with the number of clients. In other words, for any number of clients K≥1, if ${⋃}_{k}D_{k}=D^{\prime }$, where $D_{k}$ is the dataset of the client *k*, k∈{1,2,⋯,K}, then for K+1 clients, ${⋃}_{k}D_{k}=D$, such that $D^{\prime }\subset D$.

For this section, asymmetric data distribution was only due to the different sample sizes among clients but with an equal number of phishing and legitimate emails. The variation in the local data sizes was based on the maximum percentage of the variation provided by the term “var.” For example, if var=10%, then the variation of the data across the five clients was given by [−10%,−5%,0%,+5%,+10%], where −10% referred to the 10% less local data, and +10% referred to the 10% more data in the respective clients. In this case, 3606, 3806, 4008, 4208, and 4408 local data samples resided in clients labeled 1, 2, 3, 4, and 5, respectively. This way, we created a variation in the local data sizes by maintaining their overall size. In addition, var=0% represented the balanced data distribution.

RQ** 4.** *Could a client leverage FL to improve its performance?*

For this research question, we limited our experiments to THEMIS (considering both email’s header and body information) because BERT had high training/testing overhead to run up to 50 global epochs.

#### 4.2.1. Experiment 1: A Client-Level and Overall Effects of Adding One New Client in FL

In this experiment, we considered five clients in total, where the first four clients (Client 1 to Client 4) participated in the FL until 15 global epochs and trained the model collaboratively. Afterward, the fifth client only carried out the learning, and the training proceeded for the subsequent 15 global epochs (i.e., until 30 global epochs). In addition, the testing results were computed for all five clients throughout the performance evaluation process. This experiment examined how a newly joined client in FL affects the performance in phishing detection.

**Figure 6:**
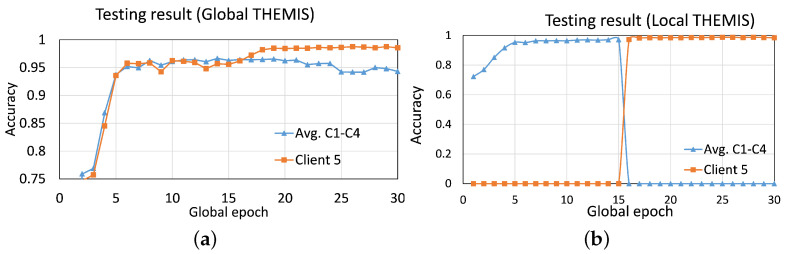
Convergence curves of (**a**) global testing accuracy, and (**b**) local testing accuracy from Experiment 1 with five clients and var=80. The first four clients trained the model until 15 global epochs, and then (only) the fifth client trained the model. C1 and C4 represent Client 1 and Client 4, respectively.

**Figure A2:**
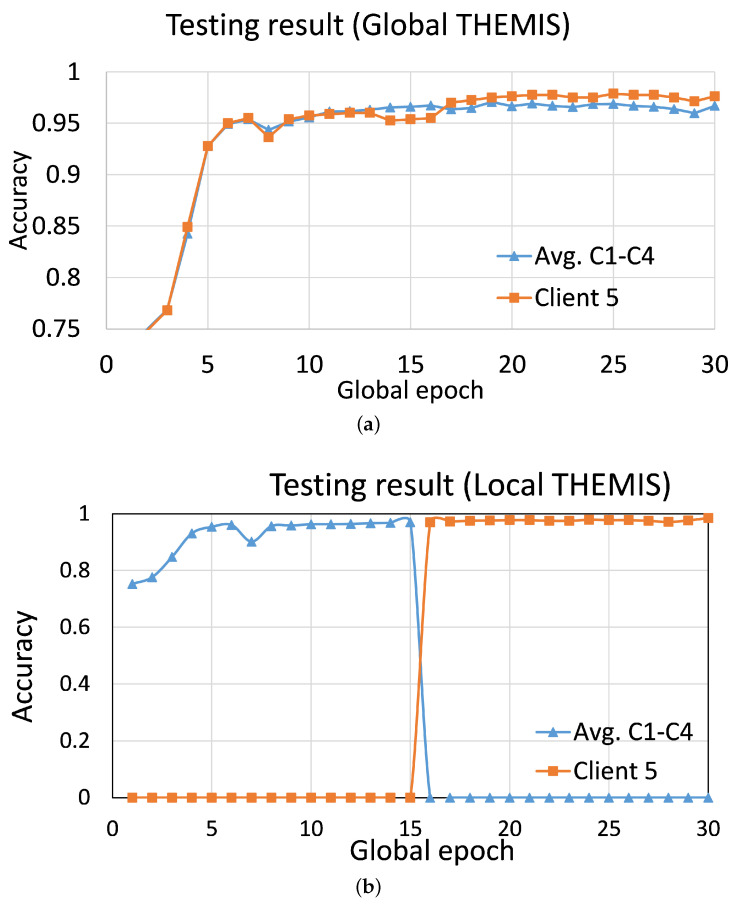
Experiment 1: Convergence curves of (**a**) global testing accuracy and (**b**) local testing accuracy from Experiment 1 with five clients and var=0. The first four clients trained the model until 15 global epochs, and then (only) the fifth client trained the model.

The experimental result depicted in Figure 6 is for the case with var=80%, which provided the variations in the sizes of the local dataset (i.e., [−80%, −40%, 0%, +40%, +80%]) to capture a practical setting among the five clients. The average global test accuracy of the first four clients was slightly higher than the fifth client (who did not participate in the learning process) until 15 global epochs. Afterward, the fifth client trained the model, so its average global testing accuracy improved by 2.98%, and FPR and FNR improved by 3.5% and 2.2%, respectively. This performance gain decreased with the lesser variation in the sizes of the local dataset; the improvements in average global test accuracy were 2.91%, 2.87%, and 2.24% with var equal to 50%, 30%, and 0%, respectively. Refer to Figure A2 in Appendix A.2 for the results with the balanced email distribution, i.e., var=0%. Overall results showed that the evolved model (after training by Client 5) was still relevant to the first four clients (Client 1 to Client 4) as their average testing results with and without Client 5 differed only nominally. Nonetheless, the fifth client boosted the accuracy of phishing detection in its local dataset.

#### 4.2.2. Experiment 2: A Client-Level and Overall Effects of Continuously Adding New Clients in FL

In this experiment, the learning process started with the first client, and then one new client joined continuously at an interval of 10 global epochs as the training proceeded. Refer to Table 3 for details. This experiment simulated the practical cases where more than one client (different than Experiment 1) was available with time during model training and demonstrated how the newly available clients could continue to perform FL in order to contribute accuracy improvements for phishing detection.

**Figure 7:**
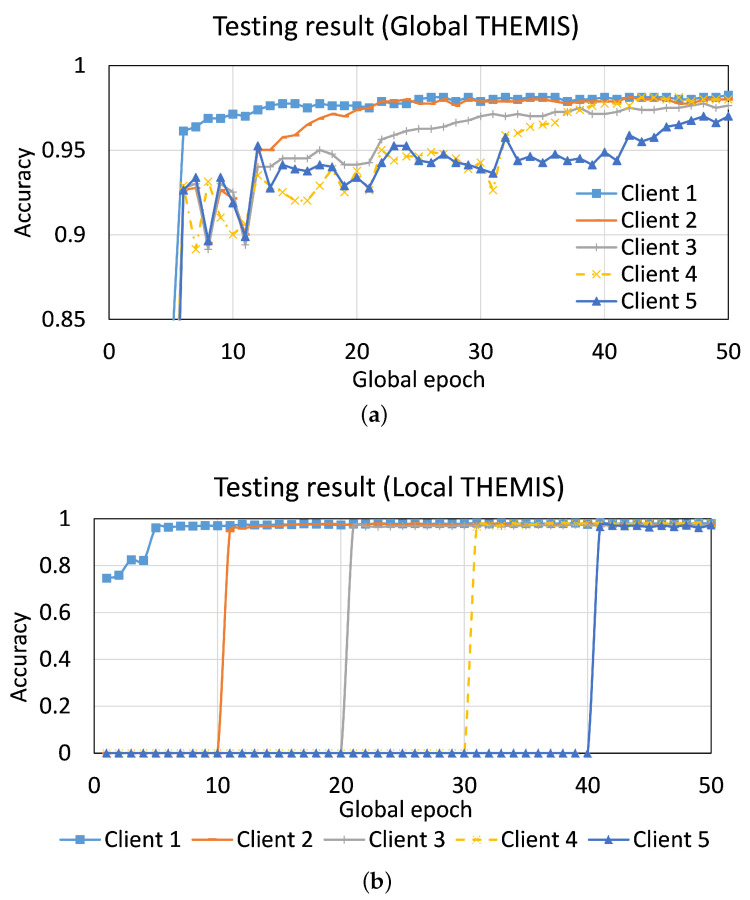
Convergence curves of (**a**) global testing accuracy, and (**b**) local testing accuracy from Experiment 2 with five clients and var=0%. The FL training started with one client, i.e., Client 1, and a new client joined the training at every 10 global epochs in a sequence from Client 2 to Client 5.

**Figure A3:**
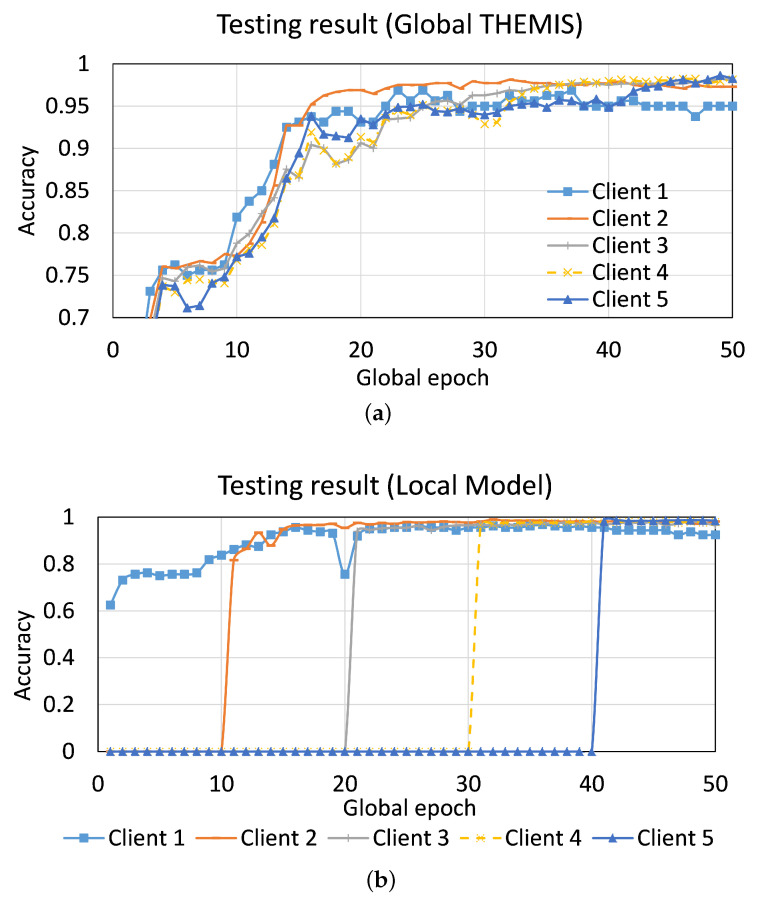
Experiment 2: Convergence curves of (**a**) global testing accuracy and (**b**) local testing accuracy from Experiment 2 with five clients and var=80. The FL training started with one client, i.e., Client 1, and a new client joined the training at every 10 global epochs in a sequence from Client 2 to Client 5.

The result depicted in Figure 7 is for the case with the same size of the local dataset among the five clients (i.e., var=0%), which were gradually added to FL, as stated in Table 3. As per expectation, the global testing accuracy improved for each client when they were added to FL. For example, the average global testing accuracy jumped by around 4.9% (corresponding to the accuracy at the global epoch 10 and 19) for Client 2 when it joined Client 1 in training the model at global epoch 10. The local testing results were carried out only when the client was involved in the model training; therefore, the local testing accuracy before a client joined the training is zero in Figure 7b. The overall performance pattern for the case with var∈{30%,50%,80%} was similar to the case with var=0%. However, we observed the dominance of late joining clients (e.g., Client 4 and Client 5) in their performance since the initial clients (e.g., Client 1) had a fewer number of samples than the late joining clients if var≠0%. Moreover, the initial client, such as Client 1, could not catch up with the performance of the late joining client, such as Client 5 (for details, refer to Figure A3 in Appendix A.2).

#### 4.2.3. Experiment 3: Benefits to the Newly Participated Client in the FL Learning Process

In this experiment, we analyzed the performance of Client 1, a newly participating client with and without leveraging FL. In the FL setup, the model was first trained by the four clients (Client 2 to Client 5) for 20 global epochs, and then the resulting model (pre-trained model) was further trained by Client 1 on its local email data samples. The dataset distribution of the clients was defined by var. On the other hand, for the case without FL, Client 1 performed CL only on its local email dataset.

**Figure 8:**
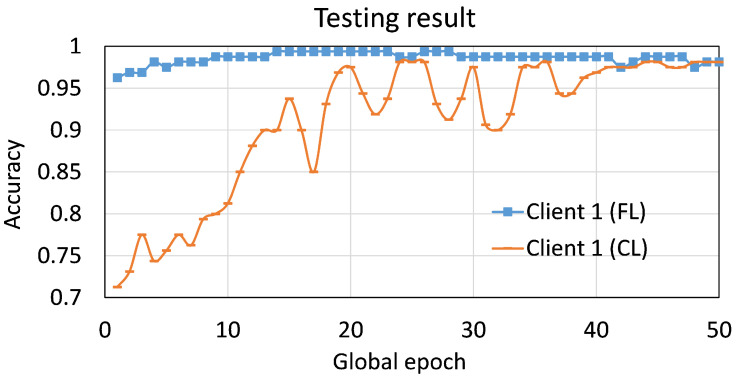
Convergence curves of (local) testing accuracy for Client 1 with and without leveraging FL. The dataset of Client 1 was based on var=80 among five clients.

The result depicted in Figure 8 is for Client 1, and its dataset was defined under five clients setup with var=80% (Client 1 had 80% fewer data samples than Client 3). Client 1 achieved a fast convergence and stable output by leveraging FL compared to its training under CL on its local dataset. However, the same final accuracy of around 98% was observed for both cases at global epoch 50. If the Client 1’s dataset was assigned based on var=0% (balanced case) with five clients, then the convergence curves for Client 1 were more stable and flat after ten global epochs; however, a fast convergence was consistently observed for this case when leveraging FL. Refer to Figure A2 in the Appendix A.2 for the results.


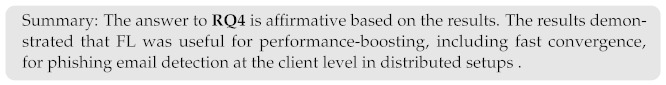


### 4.3. Distributed Email Learning under Asymmetric Data Distribution

We examined the performance of FL in an asymmetric dataset distribution mainly in two forms; (1) different sample sizes among clients (defined by var) but with an equal number of phishing and legitimate emails, and (2) same sample size but a different number of phishing and legitimate emails. This setup was not precisely a non-IID distribution, which is due to the high skewness in the number of samples and their classes present in each client’s dataset.

RQ** 5.** *How would FL perform considering asymmetric data distributions among clients due to the variations in local dataset sizes and local phishing to legitimate sample ratios?*

We performed two experiments based on the phishing to legitimate email samples (P/L) ratio among clients.

#### 4.3.1. Same P/L Ratio across Clients but Having Different Sizes of the Local Dataset

**Figure 9:**
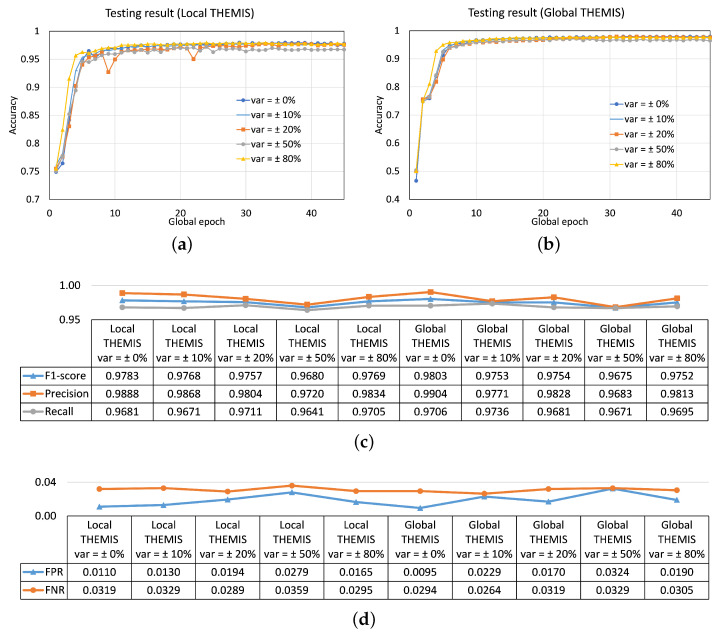
The impact of different local data sizes provided by different var among clients to the convergence in FL with five clients on testing accuracy curves of (**a**) local model and (**b**) global model. Corresponding performance metrics of the testing results at global epoch 45 in (**c**) centralized learning and (**d**) federated learning (FL) with five clients.

**Figure A5:**
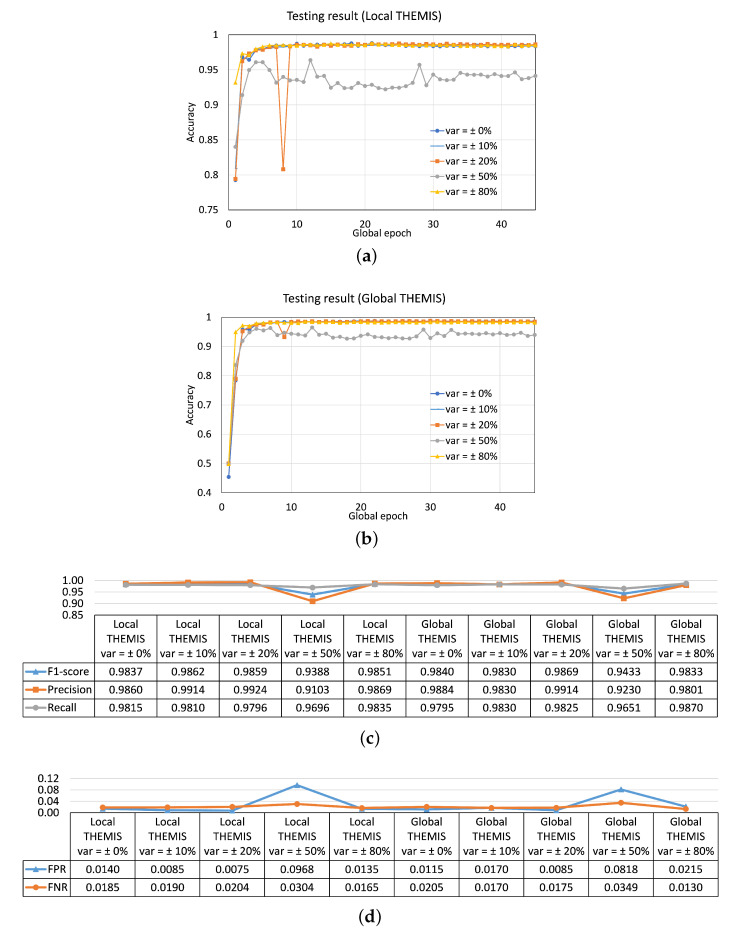
The impact of different local data sizes provided by different var among clients to their convergence in FL with two clients on testing accuracy curves of (**a**) local model and (**b**) global model. Corresponding performance metrics of the testing results at global epoch 45 in (**c**) centralized learning and (**d**) federated learning (FL) with 10 clients.

Figure 9 depicts the result for 0% (balanced), 10%, 20%, 50%, and 80% variations in the sizes of the local dataset in FL among 5 clients. In the results, the convergence of the test accuracy curves rose until global epoch 10, then remained almost flat afterward. All cases with different var maintained an overall testing accuracy of around 97% and similar FPR and FNR at global epoch 45 (Figure 9). We observed similar performance patterns for FL with 10 clients. However, with 2 clients, the test performances were improved relative to 5 or 10 clients for all cases except with var=50 (refer to Figure A5 for details). The results showed that the closeness of the performance for var=50 to other cases of var improved with an increase in the number of clients; it achieved the same performance as others for FL with 10 clients.

For most cases, the similarity in performance despite variations in the local data sizes amongst clients indicated the FL’s resilience (primarily enabled by weighted averaging) to the data size variations.

#### 4.3.2. Different Legit Email to Phishing Email Sample Ratio across Clients but Having the Same Sizes of the Local Dataset

**Figure 10:**
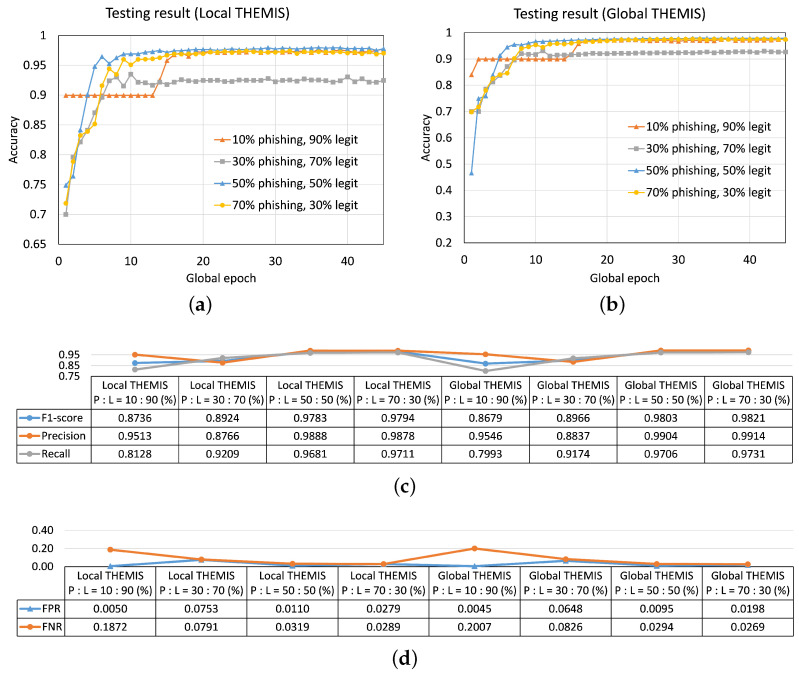
The impact of different legit email to phishing email sample ratios in the local dataset to the convergence in FL with five clients on testing accuracy curves of (**a**) local model and (**b**) global model. Corresponding performance metrics of the testing results at global epoch 45 in (**c**) centralized learning and (**d**) federated learning (FL) with five clients.

Figure 10 depicts the results for FL with 5 clients having the same size of the local dataset but all with P/L ratios of (i) 10:90 (first case), (ii) 30:70 (second case), (iii) 50:50 (third case), and (iv) 70:30 (fourth case). We chose the specific ratios for the test purpose so that the P/L ratio remained distinct. This setup was more practical than the setup with the same P/L ratio, as this had a bias in the samples. The experiments for this section had var=0. In the results, until global epoch 15, there was a difference in the performance, where the first case (i.e., 10:90 P/L ratio) with the lower phishing email samples was not performing well compared with other cases with higher phishing email samples. However, after epoch 15, all cases converged similarly to provide an overall testing accuracy of around 97% except for the second (30:70 P/L ratio) case, where the testing accuracy was around 93%. Other performance metrics are provided in Figure 10c,d.

**Figure A6:**
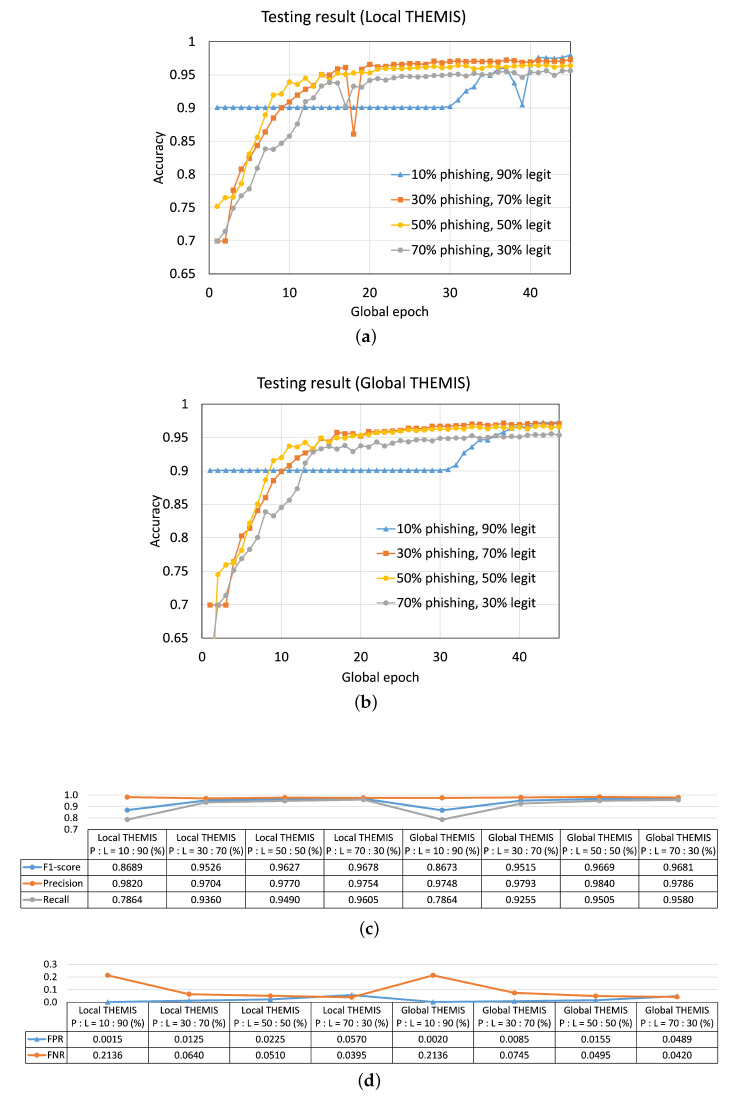
The impact of different legit email to phishing email sample ratios in the local dataset to their convergence in FL with ten clients on testing accuracy curves of (**a**) local model and (**b**) global model. Corresponding performance metrics of the testing results at global epoch 45 in (**c**) centralized learning and (**d**) federated learning (FL) with 10 clients.

Comparing the results for FL with 2, 5, and 10 clients (see Figure A6 in Appendix A.4 for the results with 10 clients), they showed a jump in the testing accuracy for the first case (10:90 P/L ratio) at different global epochs; jumped at global epoch 5, 15, and 30 for FL with 2, 5 and 10 clients, respectively. The reasons behind these jumps were unclear. Overall, observing at global epoch 45 for FL with 2, 5, and 10 clients, the global performance for various P/L ratios slightly decreased with the increase in phishing samples (from 10% to 70%). In practice, organizations have fewer phishing emails than legitimate ones; thus, this concern can be contained.


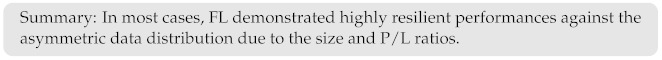


### 4.4. Distributed Email Learning under an Extreme Asymmetric Data Distribution

We considered an extreme dataset diversity among the clients. We kept our different email sources as different clients: Client 1 had IWSPA dataset, Client 2 had Enron dataset, Client 3 had Nazario dataset, Client 4 had CSIRO emails, and Client 5 had Phishbowl emails (refer to Table 1 for the size of local datasets). This setup captured the variations in the sample sizes and class types across clients.

RQ** 6.** *How would FL perform under extreme dataset diversity among clients?*

**Figure 11:**
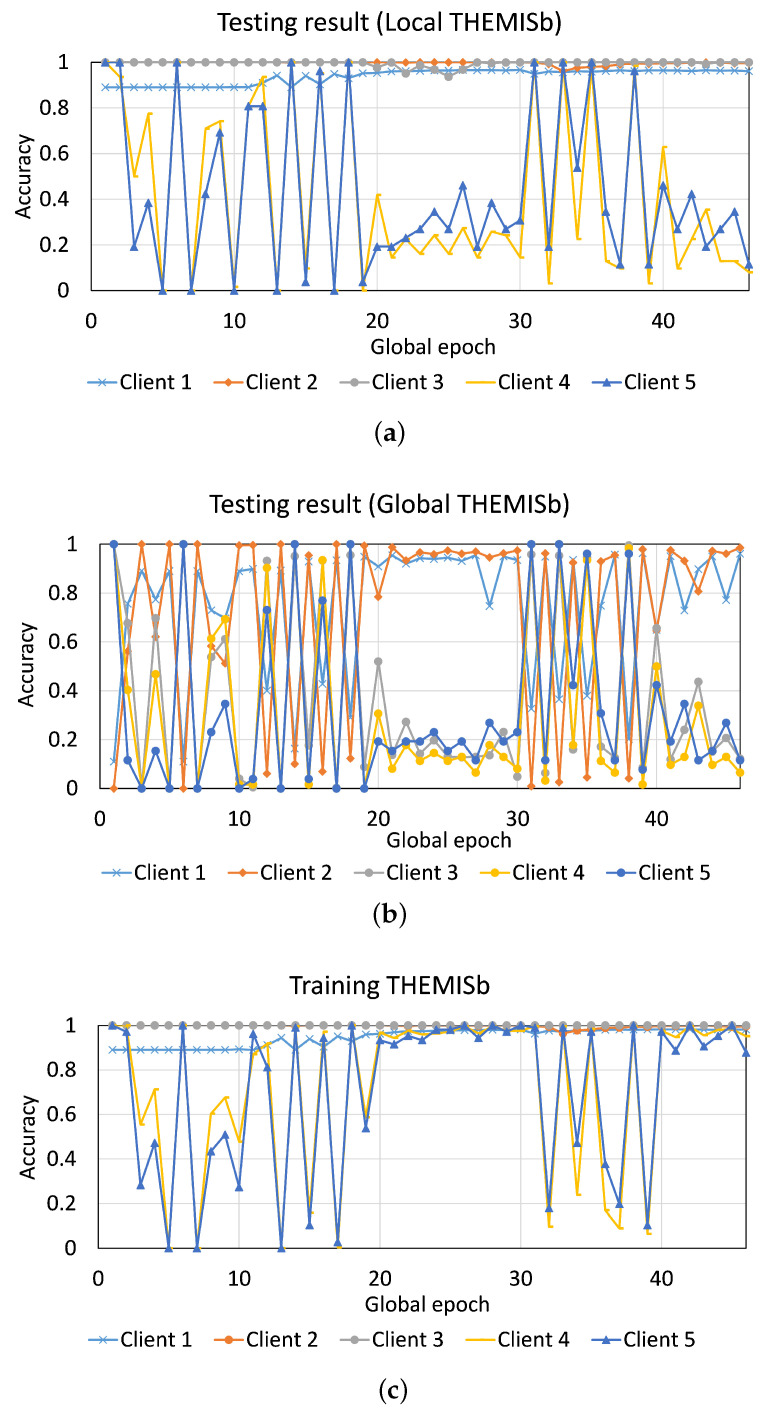
FL with five clients and THEMISb: Client-level convergence curves of (**a**) testing accuracy of the local model, (**b**) testing accuracy of the global model, and (**c**) training accuracy.

**Figure 12:**
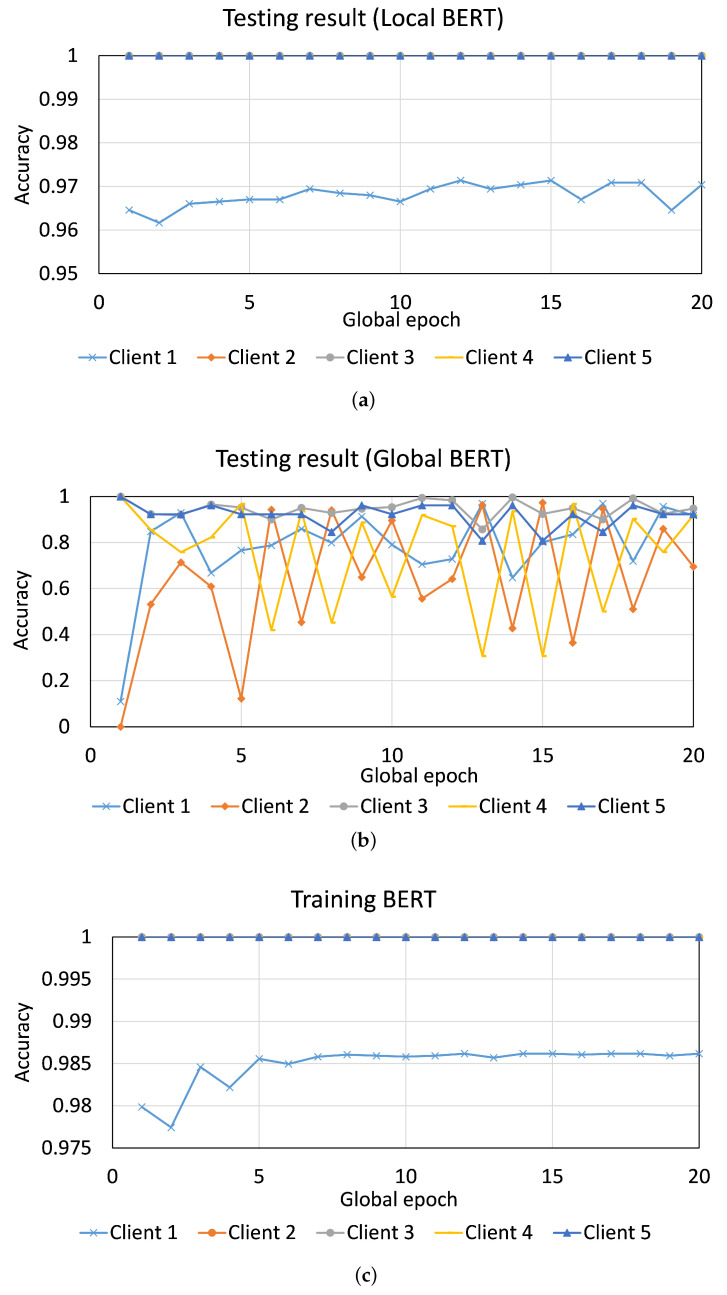
FL with five clients and BERT: Client-level convergence curves of (**a**) testing accuracy of the local model, (**b**) testing accuracy of the global model, and (**c**) training accuracy.

**Figure A7:**
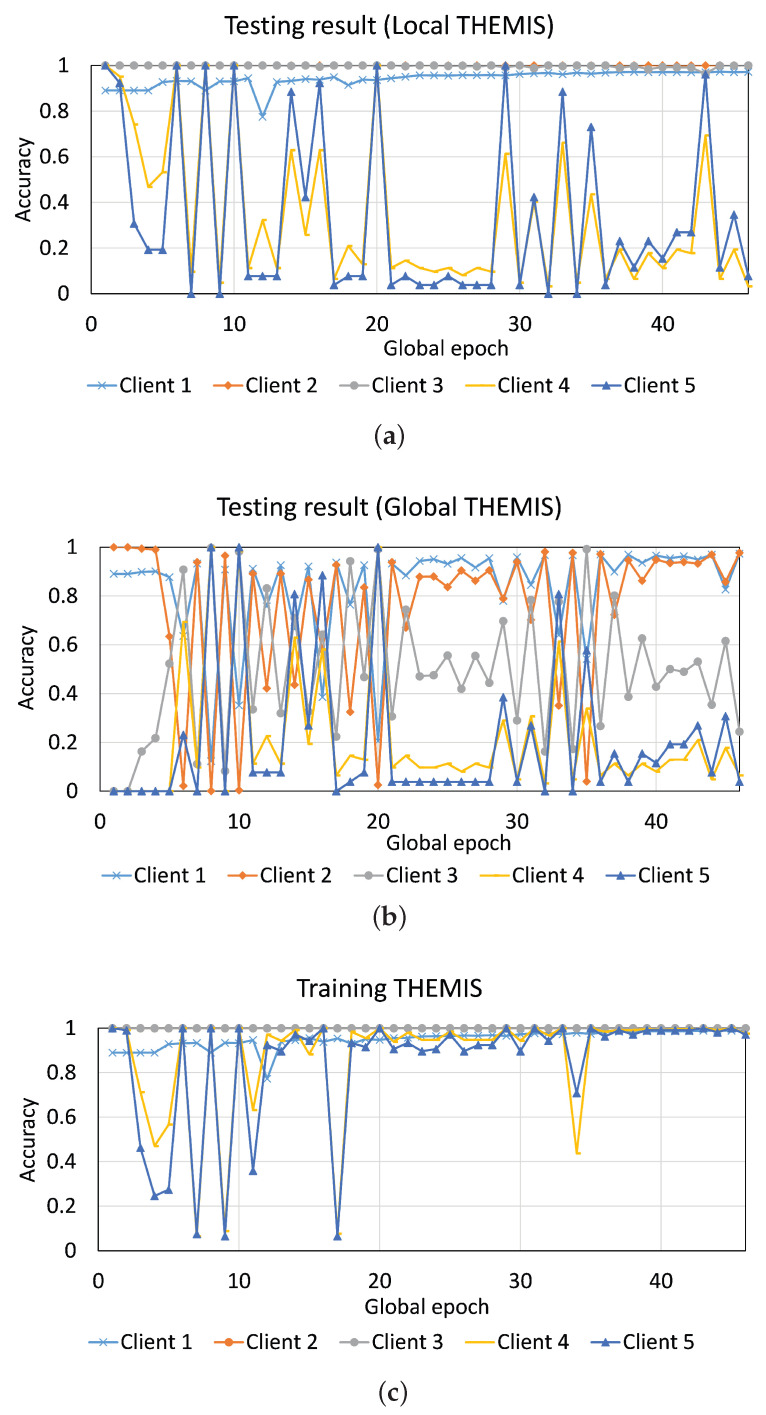
FL with five clients and THEMIS: Client-level convergence curves of (**a**) testing accuracy of the local model, (**b**) testing accuracy of the global model, and (**c**) training accuracy.

Figure 11 and Figure 12 depict the results for THEMISb and BERT in FL, where a data sample had only the email body. For THEMIS and THEMISb, clients having few samples and the latest phishing email samples, such as Client 4 and Client 5, could not collaborate effectively while training/testing and suffered from high fluctuations in their results. Moreover, Client 3, which had only phishing emails but in large numbers, also performed similarly to Client 4 and Client 5 in the global model testing. However, its local model testing result was excellent (around 99.99% accuracy). Both the THEMIS models (THEMIS and THEMISb) showed similar results. Refer to Figure A7 in the Appendix A.5 for the results of THEMIS where the data samples considered the email headers.

Unlike FL with THEMISb and THEMIS, all clients converged well during FL training with BERT and performed well in the local model testing. Moreover, Client 1 was underperforming among all, with 97% accuracy in the local model testing; others maintained an accuracy of 99.99%. However, the global testing results showed high fluctuations, specifically for Client 1, Client 2, and Client 4. Client 4 and Client 5 had relatively stable results in global model testing than other clients. THEMIS/THEMISb had a different result in this case. The problem with THEMIS/THEMISb in FL can be contained by allowing the clients to train their local models for a longer time and keep either the local or global model based on their performance for the deployment.


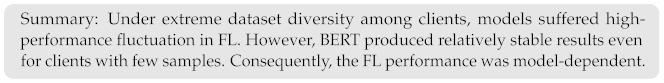


## 5. Related Works

### 5.1. Centralized Learning in Phishing Detection

A centralized email analysis based on AI-based methods for phishing detection has been explored for a long time. Conventional ML-based techniques, such as decision trees, logistic regression, random forests, AdaBoost, and support vector machines, were analyzed in phishing detection [8,39,40,41,42,43,44]. These techniques were based on feature engineering, which requires in-depth domain knowledge and trials. On the other hand, DL-based methods included deep neural networks [45], convolutional neural networks (CNNs) [5], deep belief networks [46], bidirectional LSTM with supervised attention [47], and recurrent convolutional neural networks [6]. These works were primarily based on natural language processing techniques for phishing detection. While most existing works had focused on effectively detecting general phishing emails, few considered specialized phishing attacks, including spear phishing attacks [48] and business email compromise attacks [49] in specific contexts. Despite the usefulness, all the above works operated under a setting where emails must be centralized for analysis, and, thus, they did not provide privacy protection for email datasets.

### 5.2. Cryptographic Deep Learning Training

There have been attempts at cryptographic approaches for supporting DL model training over encrypted data, which can be applicable for phishing email detection while preserving privacy. The first system design for privacy-preserving neural network training was SecureML [50]. In this system, multiple data providers secretly shared their data among two cloud servers, which then conducted the training procedure over the secret-shared data. This work relied on secure computation techniques, e.g., secret sharing, and garbled circuits, to design a secure two-party computation protocol, allowing two cloud servers to compute the linear operations (addition and multiplication) in the ciphertext domain as the non-linear activation functions. Later, a design that works in the three-server model was proposed [51]. It was based on the lightweight secret-sharing technique with better performance than SecureML. This work assumed an adversary model where none of the three cloud servers would deviate from the protocol. The work in [52] also operated under a similar three-server setting yet achieved more robust security against malicious adversaries who deviated arbitrarily. This line of work presented valuable research endeavors to enable deep neural network training over encrypted data. Yet, it had to rely on additional architectural assumptions (i.e., non-colluding cloud servers) and incurred substantial performance overheads (up to orders of magnitude slower) compared to the plain text baseline.

### 5.3. Federated Learning

FL is attractive, especially when the data are sensitive, in the financial sector (banks) and the medical sector (hospitals) [18]. There have been several works in FL, though none specifically addressing phishing email detection. Some works included when Google used FL for next-word prediction in a virtual keyboard for smartphones words [53], Leroy et al. applied FL for speech keyword spotting [54], Gao et al. [55] proposed to use FL to train a joint model over heterogeneous ECG medical data to preserve the data privacy of each party, and Yang et al. [17] applied FL to detect credit card fraud. The common model aggregation method in FL is federated averaging (FedAvg) [56]. In addition, FedCurv [57], FedProx [58], and FedAwS [59] have been proposed for better results, specifically when the data distribution among the clients is imbalanced or non-identical distributions providing heterogeneity in data in FL.

## 6. Discussion and Future Work

This paper was the first step in federated email learning for phishing detection. Though our results demonstrated its benefits and performances, FL needs more studies, specifically for its robustness under security attacks.

### 6.1. Improving Federated Learning Performance in Phishing Email Detection

This paper considered the federated averaging (FedAvg) algorithm for model aggregation in FL. However, it was reported in the literature that FedAvg could have a hugely detrimental effect on the model’s performance because many variants of the model weights only differ in the ordering of parameters, such as in neural networks [60]. In addition, the personalization of the model may cause the deterioration of the model performance due to the FedAvg [61]. Personalization means that the local model may fit nicely for some but not all devices. This means some devices will have outstanding performance, and otherwise for others (the case for Client 4 and Client 5 with the THEMIS model in an extremely asymmetric data distribution). With more users, the effect of personalization is more significant, resulting in a low-performance global model. This effect can be mitigated using model agnostic meta-learning [61] to improve the global model so that it can fit well for most users and has a faster convergence. As a side effect, the local test accuracy, due to the less personalized effect, is less likely for some devices to have excellent local model performance. Therefore, in phishing detection, studying other aggregating methods such as FedCurv, FedProx, and FedAwS may be required to improve the results further. In addition, studies with more models, a large corpus of email data, and their comparative analysis in FL are left as the subsequent work.

### 6.2. Federated Learning, Privacy, and Security Attacks

The main challenge for deploying FL in phishing detection would be the possibility of security and privacy attacks in FL, observed in datasets other than phishing emails. Moreover, privacy could be leaked due to the inference attack [62], while security attacks can occur due to backdoor attacks via data poisoning or parameter tampering [63]. Corresponding countermeasures, such as input filters, unlearning, strong intentional perturbation-based Trojan attack detection, and dynamic client allocation mechanism [16,64,65,66], can be deployed to mitigate such attacks. However, future works are required to analyze their implications in phishing detection.

In addition, the security analysis of federated learning is required considering various attacks, including an active attack, where an attacker can obtain the communicated plaintexts, and a passive attack, where an attacker knows the communicated ciphertexts [67].

### 6.3. Data and Model Privacy

FL is a privacy-by-design approach; however, FL alone cannot guarantee data privacy. So, there are various other techniques, such as homomorphic encryption [68] (a cryptographic approach) and differential privacy [69], used along with FL for guaranteed and provable privacy, respectively. However, homomorphic encryption increases computational overhead, and differential privacy degrades the performance as a trade-off. Integrating these techniques into FL in phishing detection remains another research avenue.

Overall, data and model privacy analyses in federated learning phishing email detection are important research aspects. In this regard, we can consider adversaries to be either a central server (e.g., cloud server) or clients (e.g., data owner) or model users/requesters during the training and inference phases [70].

## 7. Conclusions

This work took the first step to implement federated learning (FL) for phishing email detection in collaborative distributed frameworks. FL did not require sharing email data, enabling multiple organizations (providing email data) to participate in training deep email anti-phishing models. These models usually had high detection but required huge amounts of email data; thus, FL enabled their training.

Built upon the best deep learning model relevant to email phishing detection, namely bidirectional encoder representations from transformers (BERT) and THEMIS/THEMISb, our analysis under FL demonstrated promising results. For an overall analysis, our studies addressed six research questions related to balanced and asymmetrical data distribution among clients, scalability, communication overhead, client-level perspectives in FL, and FL performance in asymmetric data distribution and extreme dataset diversity among clients. Our results demonstrated that FL achieved comparable performance to centralized learning under balanced data distribution, with THEMIS achieving 97.9% test accuracy for FL with five clients at epoch 45 and BERT achieving 96.1% test accuracy for FL with five clients at epoch 15. FL was also resilient to asymmetrical data distribution scenarios, with THEMIS performing well under different local dataset sizes and phishing to legitimate sample ratios. However, forming a best-performing global model for all clients under FL with extreme dataset diversity is not straightforward, and local and global performances are model dependent—BERT was relatively more stable than THEMIS/THEMISb. There was communication overhead, but that was not a particular concern for organizational-level participants.

## Figures and Tables

**Table 1:** The number of email samples extracted from various sources.

Source	Phishing (P)	Legitimate (L)	P + L
IWSPA-AP	1132	9174	10,306
Nazario	8890	0	8890
Enron	0	4279	4279
CSIRO	309	0	309
Phisbowl	132	0	132
Total	10,463	13,453	23,916

**Table 2:** Dataset setup for empirical analysis based on six research questions (RQs).

Research Questions	Data Distribution among Clients		Data Source				
	Balanced	Asymmetric	IWSPA-AP	Nazario	Enron	CSIRO	Phishbowl
RQ1	✓	✗	✓	✓	✓	✗	✗
RQ2	✓	✗	✓	✓	✓	✗	✗
RQ3	✓	✗	✓	✓	✓	✗	✗
RQ4	✓	✓	✓	✓	✓	✗	✗
RQ5	✗	✓	✓	✓	✓	✗	✗
RQ6	✗	✓	✓	✓	✓	✓	✓

**Table 3:** Experimental steps for Experiment 2.

Round	Involvement of Clients
0 to 9	Only the first client.
10 to 19	Only the first and second clients.
20 to 29	Only the first, second, and third clients.
30 to 39	First, second, third, and fourth clients.
40 to 50	All five clients.

Note: The local and global test accuracy were measured for all clients throughout the process.

**Figure A4:**
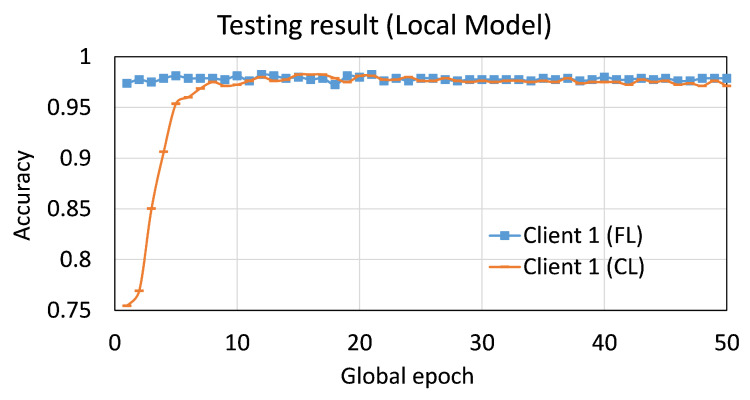
Experiment 3: Convergence curves of the (local) testing accuracy for Client 1 with and without leveraging FL. The dataset of Client 1 was based on var=0 among five clients.

## Data Availability

The data presented in this study are available on request from the corresponding author. The data are not publicly available due to privacy.

## Abbreviations

The following abbreviations are used in this manuscript:

DL	Deep learning
RCNN	Recurrent convolutional neural network
GDPR	General data protection regulation
FL	Federated learning
NLP	Natural language processing
BERT	Bidirectional encoder representations from transformers
